# Risk factors for retear of large/massive rotator cuff tears after arthroscopic surgery: an analysis of tearing patterns

**DOI:** 10.1186/s13018-017-0643-7

**Published:** 2017-09-25

**Authors:** Hisao Shimokobe, Masafumi Gotoh, Hirokazu Honda, Hidehiro Nakamura, Yasuhiro Mitsui, Tatsuyuki Kakuma, Takahiro Okawa, Naoto Shiba

**Affiliations:** 1 Department of Orthopaedic Surgery, Kurume University School of Medicine, 67 Asahi-machi, kurume, Fukuoka, 830-0011 Japan; 2 Department of Statistics, Kurume University School of Medicine, 67 Asahi-machi, kurume, Fukuoka, 830-0011 Japan; 3 Department of Orthopaedic Surgery, Kurume University Medical Center, 155-1 Kokubu-machi Kurume, Fukuoka, 839-0863 Japan

**Keywords:** Tearing pattern, Arthroscopic rotator cuff repair, Postoperative retear

## Abstract

**Background:**

Previous studies have evaluated the risk factors for retear of large/massive rotator cuff tears (RCTs) that were treated arthroscopically; however, most studies did not evaluate tear patterns. The present study hypothesized that postoperative risk factors are affected by the tearing patterns in large/massive cuff tears in patients undergoing arthroscopic rotator cuff repair (ARCR).

**Methods:**

One hundred fifty patients with large/massive cuff tears underwent ARCR at our institution. Of these, 102 patients were enrolled in this study, with an average symptom duration of 36.3 ± 43.9 months and average age of 63.9 ± 9.4 years. According to the arthroscopic findings and magnetic resonance imaging (MRI), the 102 patients were divided into three groups based on the tendon location: anterosuperior tears (*N* = 59, group AS), posteosuperior tears (*N* = 21, group PS), and anteroposterior-extending tears (*N* = 22, group APE). Functional outcome was evaluated preoperatively and postoperatively using the Japanese Orthopedic Association (JOA) score and the University of California, Los Angeles (UCLA) score. Retear was evaluated with MRI at a minimum of 1 year after surgery, using Sugaya’s classification; Types IV and V were considered postoperative retears. Factors affecting postoperative retear were examined with univariate and multivariate analyses.

**Results:**

JOA/UCLA scores significantly improved postoperatively in the three groups (*P* < 0.01 for all). Postoperative retear was noted in 26 of 102 patients (25.5%) in this series: 10 patients in group AS (16.9%), 9 in group PS (42.9%), and 7 in group APE (31.8%). The retear rate was significantly higher in group PS than in the other two groups (*P* = 0.02). Multivariate analysis showed that decreased preoperative active external rotation range was a unique risk factor for postoperative retear in the PS and APE groups (95% confidence interval: 0.02–0.18, cut-off value: 25°, with an area under the curve of 0.90, *P* = 0.0025).

**Conclusions:**

Although multivariate analysis failed to detect significant risk factor for retear in patients with anterosuperior large/massive cuff tears who undergo ARCR, it demonstrated that active external rotation less than 25° before surgery is a significant risk factor in those with posterosuperior large/massive tears. This study may help surgeons understand the results of arthroscopic surgery in patients with large/massive tears.

## Background

Arthroscopic rotator cuff repair (ARCR) produces good clinical results, although retear is a significant concern after surgery. Compared with small- and middle-sized rotator cuff tears (RCTs), the retear rate is relatively high in large and massive tears, even if the tear is completely covered during surgery [1–4]; some authors reported that the retear rate was 40–94% in these tears [3–6].

A number of studies have consistently sought to determine the risk factors for postoperative retear in large and massive cuff tears. For example, a recent systematic review reported that the risk factors for retear after ARCR in RCTs included age, tear size, fatty degeneration (FD), the number of tendons involved, acromiohumeral interval, surgical technique, and bone mineral density [7]. One study used a multivariate regression analysis to demonstrate that preoperative FD of the infraspinatus was the most independent predictor of retear in large and massive RCTs in patients who underwent ARCR [8]. Kim et al. [9] reported that the extent of retraction was importantly associated with retear after surgery.

Based on the tendon location involved, large and massive RCTs are classified into three types: anterosuperior tears, posteosuperior tears, and anteroposterior-extending tears. However, previous studies collectively examined large and massive RCTs without sub-dividing the tear pattern as described above. Therefore, the purposes of the present study were to evaluate risk factors affecting postoperative retear in each group. We hypothesized that in large and massive tears, the risk factors for postoperative retear differ among the groups, when sub-divided by the tear pattern.

## Methods

The patients provided informed consent, and this retrospective study was approved by the authorized institutional review board at the Ethical Committee of Kurume University (#12333).

### Patients

Between April 2005 and August 2013, 150 patients with cuff tears defined as large or massive [6] underwent ARCR in our institution. The inclusion criteria were (1) individuals who had large or massive rotator cuff tears that repaired completely during surgery, (2) those who were available for evaluation of function and magnetic resonance imaging (MRI) preoperatively and at a minimum of 1 year after surgery, and (3) those who underwent an appointed postoperative rehabilitation program. The exclusion criteria were (1) individuals with advanced glenohumeral arthritis or fractures around the shoulder, (2) those who underwent open repair, partial repair, revision surgeries, or any previous shoulder surgery, (3) those who had MRI film without the “Scapula - Y” view on the sagittal-oblique plane, (4) those who refused to undergo postoperative clinical assessment and MRI, and (5) those who had preoperative stiffness that showed less than 100° in passive elevation or 10° in external rotation [10]. Consequently, 102 patients were enrolled in this study.

According to the arthroscopic and MRI findings, the 102 patients were divided into three groups based on the tendon location: anterosuperior tears [11] in the subscapularis and the supraspinatus, in which the tear extended from the lesser tuberosity to the superior facet (*N* = 59, group AS); posteosuperior tears in the supraspinatus and the infraspinatus/teres minor, in which the tear extended from the superior facet to the middle or inferior facet (*N* = 21, group PS); and anteroposterior-extending tears, in which the tear extended from the lesser tuberosity to the middle or inferior facet (*N* = 22, group APE). When large or massive tears were evaluated according to the classification of DeOrio and Cofield [12], there were 56 patients with a large tear (94.9%) and 3 patients with a massive tear (5.1%) in Group AS, 19 patients with a large tear (90.5%) and 3 patients with a massive tear (9.5%) in group PS, and 15 patients with a large tear (58.2%) and 7 patients with massive tears (31.8%) in group APE. There was no statistical difference in demographic data among the three groups, except for the incidence of hypertension and distribution of massive tears. Details of the patients’ characteristics are shown in Table 1.

**Table 1:** Patient demographic data

	Group AS (*N* = 59)	Group PS (*N* = 21)	Group APE (*N* = 22)
Age (years)	62.8 ± 10.6 (39–82)	64.9 ± 8.8 (43–78)	66.4 ± 5.8 (54–76)
Sex: male (%)/female (%)	30 (50.1%)/29 (49.1%)	13 (62%)/8 (38%)	13 (59%)/9 (41%)
Side: right (%)/left (%)	43 (72.9%)/16 (27.1%)	11 (52.4%)/10 (47.6%)	19 (86.4%)/3 (13.6%)
Symptom duration (week)	30.8 ± 30.3 (4–156)	53.9 ± 69.9 (4–275)	34.1 ± 40.9 (2–150)
Trauma (%)	34 (57.6%)	16 (76.2%)	12 (54.5%)
Complication			
Diabetes Mellitus (%)	7(11.9%)	16 (76.2%)	2 (9.0%)
Hypertension (%)	16(27.1%)	* 3 (14.3%)	10(45.5%)
De Orio and Cofield’s classification			
Large (%)	56 (94.9%)	19 (90.5%)	15 (58.2%)
Massive (%)	3 (5.1%)	2 (9.5%)	*7 (31.8%)
Surgical procedure			
Suture bridge (%)	40 (67.8%)	14 (66.7%)	17 (77.3%)
Simgle row (%)	14 (23.7%)	4(19.0%)	2 (9.0%)
Double row (%)	5 (8.5%)	3 (14.3%)	3 (13.7%)
LHB tenotomy (%)	29 (49.2%)	6 (28.6%)*	13 (59.0%)

Data are presented as mean ± standard deviation unless otherwise indicated LHB, long head biceps

*Statistically significant (*P* < .05) among the three groups

### Surgical procedure

Arthroscopic surgery was indicated when successful non-operative treatment, such as anti-inflammatory medications, physical therapy, subacromial or glenohumeral injections of corticosteroids or hyaluronic acid, or activity modification, was not achieved within 3 months of the first visit.

ARCR was conducted with the patient in the beach chair position under general anesthesia. At first, a glenohumeral examination was performed through a posterior portal and then transferred to the subacromial bursa. After making a lateral portal, we identified the ruptured tendon edge and evaluated its flexibility by grasping the tendon and reducing the edge to the original footprint. Capsular release was conducted from the anterior, anterolateral, or posterolateral portal; if needed, tenotomy of the long head biceps was performed. The method of cuff repair was selected based on the operative findings, tendon mobility, and tear condition with a single-row, double-row, or suture bridge technique (Table 1).

### Rehabilitation protocol

Postoperatively, the patient’s arm was fixed into a sling with an abduction pillow. Passive range of motion (ROM) exercises of the shoulder were conducted 4 days after surgery. Active ROM exercises and isometric exercise were started 6 weeks after surgery, and isotonic muscle strengthening exercises began 12 weeks after surgery.

### Evaluation of functional outcome

Functional outcome was evaluated preoperatively and postoperatively. The visual analog scale was used to measure pain (rest, night, and motion), the range of active motion was measured with a goniometer, muscle strength was measured with a handheld dynamometer (Micro FET2, Hoggan Health Industry, West Jordan, UT, USA), Japanese Orthopedic Association (JOA) score, and the University of California, Los Angeles (UCLA) score. An independent physiotherapist who was blinded to this study performed physical tests.

### Evaluation of structural outcome

Acromiohumeral distance was evaluated, using the Oizumi classification [13], on plain radiographs that were taken with the patients standing and their arm held in a neutral position.

Tear length and width were measured on MRI using the protocol of Davidson et al. [14] FD of the supraspinatus, the infraspinatus/teres minor, and the subscapularis were evaluated on the most lateral oblique sagittal T2-weighted MRI with the scapular body (the “Y-view”) [15, 16], using both Goutallier classification system and ImageJ [14]. The infraspinatus and teres minor were combined into a single measurement, because their borderline was not always clearly confirmed [17]. Muscle atrophy (MA) was evaluated using the relative ratio of the cross-sectional area of the subscapularis, supraspinatus, and infraspinatus/teres minor muscle belly to that of the supraspinatus fossa. For this measurement, we used ImageJ using the protocol of Nakamura et al. [18]

Retear of the rotator cuff was evaluated using Sugaya’s classification [19]: type I, sufficient thickness and evenly low intensity; type II, sufficient thickness and heterogeneous high intensity; type III, repaired cuff tear that kept its continuity but had insufficient thickness; type IV, minor discontinuity and the torn area was minimal in the sagittal plane; and type V, major discontinuity and torn area spread in the sagittal plane. Patients with types IV and V were admitted with postoperative retear [20]. An experienced, orthopedics-trained radiologist who was blinded to the study reviewed these images.

### Statistical analysis

The statistical analysis was performed with JMP11 software (SAS, Cary, NC, USA). The Kruskal-Wallis test or *χ* ^2^ test was used to compare the continuous or nominal variables in demographics and functional and structural outcomes among the three groups. A Wilcoxon test was used for comparing the preoperative and postoperative functional outcomes in each group. Spearman’s *ρ* was calculated to observe the nonparametric correlation of structural outcomes and clinical outcomes. The correlation between the data evaluated with Goutallier’s classification and ImageJ was examined with Spearman’s correlation coefficient. For identifying the risk factors for retear after surgery, univariate analysis was first performed in each group; then, multivariate logistic regression was performed using a step wise manner. A receiver-operating curve was calculated to detect the cut-off value when significance was noted in the multivariate analysis. The level of significance was defined for all calculations as *P* < .05. Data are expressed as a mean value with standard deviation.

## Results

### Preoperative and postoperative functional outcome

Preoperative JOA scores significantly improved from 57.9 ± 19.9 points preoperatively to 87.4 ± 10.0 points postoperatively in group AS, from 56.9 ± 15.4 points to 89.9 ± 6.6 points in group PS, and from 61.7 ± 7.5 points to 83.0 ± 11.4 points in group APE. Consistently, UCLA scores significantly improved from 18.2 ± 5.0 points preoperatively to 28.3 ± 7.2 points postoperatively in Group AS, from 17 ± 4.7 points to 29.4 ± 3.8 points in group PS, and from 15.9 ± 4.0 points to 28.3 ± 5.9 points in group APE. There were no significant differences of postoperative JOA/UCLA scores among the groups. The clinical outcomes scores are shown in Table 2.

**Table 2:** Preoperative and postoperative clinical outcome in three groups

	Group AS (*N* = 59)	Group PS (*N* = 21)	Group APE (*N* = 22)
JOA score			
Preoperative	57.9 ± 19.9(0–89.5)	56.9 ± 15.4(12–82)	61.7 ± 7.5 (46–78.5)
Postoperative	87.4 ± 10.0(65–100)	89.9 ± 6.6 (80.5–99.5)	83.0 ± 11.4 (60–96)
*P* value	< 0.001	< 0.001	< 0.001
UCLA score			
Preoperative	18.2 ± 5.0 (7–31)	17 ± 4.7 (9–26)	15.9 ± 4.0 (8–23)
Postoperative	28.3 ± 7.2 (20–35)	29.4 ± 3.8 (22–35)	28.3 ± 5.9 (17–35)
*P* value	< 0.001	< 0.001	< 0.001

*JOA* Japanese Orthopedic Association, *UCLA* University of California, Los Angeles

Rest, motion, and night pain levels in the three groups were significantly improved postoperatively, except for rest pain in group APE, but it tended to have significance (*P* = 0.06). Most of the parameters in ROM and muscle strength in the three groups significantly improved or tended to have statistical significance after surgery.

### Preoperative structural outcome

In the Oizumi classification, Class 0 was observed in 19, 6, and 3 patients; Class I in 30, 7, and 8 patients; Class II in 8, 7, and 4 patients, and Class III in 2, 1, and 5 patients in Group AS, PS, and APE, respectively. Only two patients in group APE had Class IV.

The average retraction of the torn tendon was 29.1 ± 6.2 mm in group AS, 31.2 ± 10.4 mm in group PS, and 37.1 ± 8.7 mm in group APE. The extent of the retraction was significantly larger in group PS and APE than group AS (*P* = 0.02). The average width of the torn tendon was 34.7 ± 66.0 mm in group AS, 37.2 ± 10.4 mm in group PS, and 45.7 ± 89.8 mm in group APE. The extent of the width was significantly larger in group PS and APE than in group AS (*P* = 0.002).

The average MA in group AS was 246.5 ± 83.5% in the subscapularis, 76.2 ± 19.8% in the supraspinatus, and 220 ± 51.9% in the infraspinatus/teres minor. For group PS, MA was 220.3 ± 67.4% in the subscapularis, 78.9 ± 18.6% in the supraspinatus, and 183.4 ± 38.5% in the infraspinatus/teres minor; for group APE, MA was 252.1 ± 82.4% in the subscapularis, 71.3 ± 16% in the supraspinatus, and 187.4 ± 54.8% in the infraspinatus/teres minor.

The average FD, measured with Image J, for group AS was 5.35 ± 8.25% in the subscapularis, 7.84 ± 10.24% in the supraspinatus, and 3.35 ± 4.92% in the infraspinatus/teres minor; for group PS, FD was 3.5 ± 5.2% in the subscapularis, 11.42 ± 10.15% in the supraspinatus, and 8.52 ± 9.23% in the infraspinatus/teres minor; and for group APE, FD was 6.0 ± 7.23% in the subscapularis, 12.2 ± 9.7% in the supraspinatus, and 6.05 ± 5.2% in the infraspinatus/teres minor.

A low-grade Goutallier stage (stages 0 to 2) was seen in over 80% patients in all three groups. A high-grade Goutallier stage (stages 3 and 4) in group AS was seen in the subscapularis of two patients, supraspinatus of nine patients, and infraspinatus/teres minor of two patients; in Group PS, in the subscapularis of no patients, supraspinatus of three patients, and infraspinatus/teres minor of three patients. In Group APE, a high-grade Goutallier stage was seen in the subscapularis of two patients, supraspinatus of five patients, and infraspinatus/teres minor of three patients. The global fatty degeneration index (GFDI) was 1.02 ± 0.62 in Group AS, 1.36 ± 0.5 in group PS, and 1.29 ± 0.5 in group APE. There was no significant difference among the three groups in GFDI (Table 3).

**Table 3:** Preoperative structural outcome in three groups

	Group AS (*N* = 59)	Group PS (*N* = 21)	Group API: (*N* = 22)
Oizumi classification			
Grade O	19 (32.2%)	6(28.6%)	3 (13.6%)
Grade I	30 (50.8%)	7 (33.3%)	8 (36.4%)
Grade II	8 (13.6%)	7 (33.3%)	4(18.2%)
Grade III	2 (3.4%)	1 (4.8%)	5 (22.7%)
Grade IV	0 (0%)	0 (0%)	2 (9.1%)
Retraction (mm)	29.1 ± 6.2	31.2 ± 10.4	37.1 ± 8.7
Width (mm)	34.7 ± 6.0	37.2 ± 10.4	45.0 + 9.8
Muscle atrophy (%)			
SSC	246.5 ± 83.5	220.3 ± 67.4	252.1 ± 82.4
SSP	76.2 ± 19.8	78.9 ± 18.6	71.3 ± 16
ISP/TM	220.4 ± 51.9	183.4 ± 38.5	187.4 ± 54.8
Fatty degeneration (%)			
SSC	5.35 ± 8.25	3.5 ± 5 20	6.0 ± 7.23
SSP	7.84 ± 10.24	11.42 ± 10.15	12.2 ± 9.7
ISP/IM	3.35 ± 4.92	8.52 ± 9.23	6.05 ± 5.2
Goutallier classification SSC			
Stage 0	24 (40.7%)	9 (42.9%)	6 (27.3%)
Stage 1	26 (44.1%)	8 (38.1%)	5 (22.7%)
Stage 2	7 (11.9%)	4 (19.0%)	9 (40.9%)
Stage 3	2 (3.4%)	0 (0%)	1 (4.5%)
Stage 4	0 (0%)	0 (0%)	1 (4.5%)
Goutallier classification SSP			
Stage 0	13 (22%)	2 (9.5%)	2 (9.0%)
Stage 1	16 (27.1%)	5 (23.8%)	5 (22.7%)
Stage 2	21 (35.6%)	11 (52.4%)	10 (45.5%)
Stage 3	7 (11.9%)	2 (9.5%)	1 (4.5%)
Stage 4	2 (3.4%)	1 (4.8%)	4 (18.2%)
Goutallier classification ISP/TM			
Stage 0	28 (47.5%)	5 (22.7%)	5 (22.7%)
Stage 1	26 (44.1%)	6 (27.3)	9 (40.9%)
Stage 2	3 (5.0%)	7 (31.8%)	5 (22.7%)
Stage 3	2 (3.4%)	3 (13.6%)	1 (4.5%)
Stage 4	0 (0%)	0 (0%)	2 (9.0%)
GFDI	1.02 ± 0.62	1.36 + 0.5	1.29 ± 0.5

*SSC* subscapularis, *SSP* supraspinatus, *ISP/TM* infraspinatus/teres minor, *GFDI* global fatty degeneration index

### Postoperative structural outcome

Postoperative retear (Sugaya types IV and V) was noted in 26 of 102 patients (25.5%) in this series: 10 patients in group AS (16.9%), 9 patients in group PS (42.9%), and 7 patients in group APE (31.8%). The retear rate was significantly higher in group PS than in groups AS and APE (*P* = 0.02) (Table 4).

**Table 4:** Postoperative retear (SUGAYA’s classification)

SUGAYA	Group AS (*N* = 59)	Group PS (*N* = 21)	Group APE (*N* = 22)	Total
Type I	28 (45.8%)	8 (38%)	6 (27.3%)	
Type II	13 (22%)	2 (9.5%)	3 (13.6%)	
Type III	8 (13.6%)	2 (9.5%)	6 (27.3%)	
Type IV	8 (13.6%)	4 (19%)	3 (13.6%)	15
Type V	2 (3.4%)	5 (23.8%)	4 (18.2%)	11
Retear	10 (16.9%)	9 (42.9%)	7 (31.8%)	26 (25.5%)

### Surgical technique and postoperative retear

Suture bridge technique was performed in 71 patients: 40 in group AS;14 in group PS; 17 in group APE. Postoperative retear occurred in 16 patients (22.5%): 6 in group AS (15.0%); 4 in group PS (28.6%); 6 in group APE (35.3%).

Single-row technique was performed in 20 patients: 14 in group AS; 4 in group PS; 2 in group APE. Postoperative retear occurred in 8 patients (40.0%): 4 in group AS (28.6%); 3 in group PS (75.0%); 1 in group APE (50.0%).

Double-row technique was performed in 11 patients: 5 in group AS; 3 in group PS; 3 in group APE. Postoperative retear occurred in 2 patients (18.2%): none in group AS (0.0%); 2 in group PS (66.7%); none in group APE (0.0%). Details are shown in Table 5.

**Table 5:** Univariate analysis in three groups

	Healed (*N* = 76)	Retear (*N* = 26)	Total (*N* = 102)
Suture bridge	55 (77.5%)	16 (22.5%)	71
Group AS	34 (85.0%)	6 (15.0%)	40
Group PS	10 (71.4%)	4 (28.6%)	14
Group APE	11 (64.7%)	6 (35.3%)	17
Single row	12 (60.0%)	8 (40.0%)	20
Group AS	10 (71.4%)	4 (28.6%)	14
Group PS	1 (25.0%)	3 (75.0%)	4
Group APE	1 (50.0%)	1 (50.0%)	2
Double row	9 (81.8%)	2 (18.2%)	11
Group AS	5 (100%)	0 (0%)	5
Group PS	1 (33.3%)	2 (66.7%)	3
Group APE	3 (100%)	0 (0%)	3

### Risk factors affecting postoperative retear

First, various parameters were evaluated to determine the risk factors for retear after surgery, using univariate analysis. Retraction (*P* = 0.039), width (*P* = 0.0023), FD of the supraspinatus (*P* = 0.0043), the Goutallier classification of the supraspinatus (*P* = 0.001), and GFDI (*P* = 0.008) were significant risk factors in group AS: Preoperative active external rotation range (*P* = 0.001), preoperative muscle strength of flexion (*P* = 0.02), and FD of the infraspinatus/teres minor (*P* = 0.048) were evaluated using ImageJ in group PS. FD of the supraspinatus (*P* = 0.002) and the infraspinatus/teres minor (*P* = 0.0074) were evaluated using ImageJ and GFDI (*P* = 0.0123) in group APE. The Goutallier stage of the infraspinatus in group PS and APE was not a significant risk factor, but it tended to have statistical significance (Table 6).

**Table 6:** Correlation between active external rotation range (AERR) and its related variables

Group AS (*N* = 59)	*P* value	Group PS (*N* = 21)	*P* value	Group APE (*N* = 22)	*P* value
Retraction	0.039	Preoperative ER	0.001	SSP FD	0.002
Width	0.0023	Preoperative FLEX MS	0.02	ISP/TM FD	0.0074
SSP FD	0.0043	ISP/TM FD	0.048	GFDI	0.012
Goutallier SSP	0.001				
GFDI	0.0084	*Goutallier ISP	0.08	*Goutallier ISP	0.068

*ER* external rotation, *MS* muscle strength, *FD* fatty degeneration, *GFDI* global fatty degeneration index

*There were no significant differences or trends

**Fig. 1:**
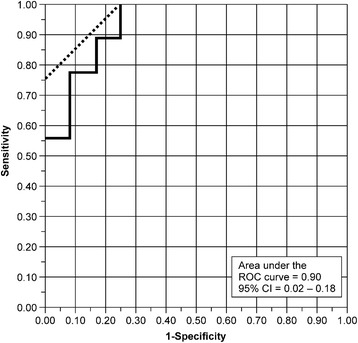
Receiver operating characteristic (ROC) curve analysis to calculate the cutoff value for preoperative active external rotation range. *AUC* area under the curve, *CI* confidence interval

Next, multivariate analysis using stepwise methods was performed. Preoperative external rotation range was the only risk factor for postoperative retear in groups PS and APE (*P* = 0.014 and *P* = 0.016, respectively). For the prediction of postoperative retear, receiver operating characteristic (ROC) curve analysis demonstrated that the cut-off value in the preoperative external rotation range was 25°, showing that the retear risk increased 2.12-fold as the preoperative external rotation range decreased by 5° (Fig. 1).

### Correlation between active external rotation range and its related variables

Since multivariate analysis showed that active external rotation range (AERR) is a unique risk factor for postoperative retear in groups PS and APE, we further evaluated the correlation between AERR and its related variables in these groups. There was statistical significance between AERR and FD, using ImageJ (*r* = −0.36, *P* = 0.04). External rotation strength (ERS) was not significant, but it showed a trend (*P* = 0.06). For FD of the infraspinatus, statistical significance was seen between the evaluation methods, using the Goutallier classification and ImageJ (*r* = 0.82, *P* < 0.0001) (Table 7).

**Table 7:** Correlation between active external rotation range (AERR) and its related variables

		Correlation coefficient (*r*)	*P* Value
Preoperative ER	Preoperative ER MS	0.3	0.06
Preoperative ER	ISP FD	−0.36	0.04
Preoperative ER	Goutallier ISP	−0.154	0.37
ISP FD	Preoperative ER MS	−0.0154	0.93
ISP FD	Goutallier ISP	0.82	< 0.001
Goutallier ISP	Preoperative ER MS	0.05	0.77

*ER* external rotation, *MS* muscle strength, *ISP* infraspinatus

## Discussion

The present study investigated the risk factors for retear after ARCR in large and massive cuff tears, dividing these tears into three groups (i.e., group AS, PS, and APE). Although univariate analysis revealed that the groups had different characteristics, step-wise multivariate analysis showed that preoperative, decreased active external rotation in group PS and APE was a unique risk factor for retear after surgery, with a cut-off value of 25°. To our knowledge, such data have not been reported.

Previous studies that used multivariate analysis demonstrated that the Goutallier stage of the infraspinatus is a risk factor for postoperative retear in large and massive tears [8, 21]. In the present study, the Goutallier stage of the infraspinatus in Group PS and APE was a significant factor for postoperative retear in univariate analysis, but not in multivariate analysis. The average Goutallier stage of the infraspinatus was relatively low (0.9) in the present study, compared with 1.2 in a study by Oh et al. [8] and 2.1 in a study by Chung et al. [21] Thus, this may partly explain why the Goutallier stage of the infraspinatus did not reach statistical significance in the present study.

FD of the infraspinatus caused postoperative retear and led to limitations of external rotation [22]. Loss of active external rotation is related to tears in the infraspinatus and teres minor [23]. In the present study, there was a significant correlation between the decrease of active external rotation range and FD, evaluated with ImageJ. Taken together, these results supported our data that decreased active external range is significantly associated with postoperative retear in patients who undergo ARCR for treatment of large or massive tears.

In the present study, multivariate analysis showed that decreased active external range before surgery was a risk factor for retear in group PS and APE. The preoperative characteristics in these two groups revealed a similar tendency, except for distribution of large or massive tears. Thus, less fatty involvement of the subscapularis in the two groups may have contributed to the similar data.

In AS cuff tears, the retear rate after surgery was reported to be 6–18% [24–26]. Consistent with these studies, our study found 10 postoperative retear cases (16.9%, *N* = 59 cases) in group AS. A previous study found that the Goutallier stage of the subscapularis was associated with postoperative retear in AS cuff tears [25], while the Goutallier stage of the supraspinatus was responsible for retear after ARCR in the present study. A high-grade Goutallier stage was found in the supraspinatus in nine cases (15.3%) and in the subscapularis in two cases (3.4%) in group AS. Thus, this might have affected our data. Although univariate analysis in the present study showed a certain risk for postoperative retear in anteroposterior cuff tears, no significant factors were noted in the multivariate analysis. Studies on these points are now underway at our institution.

Although most tears occurred in the supraspinatus tendon, tearing in this tendon did not influence retear after surgery in group PS and APE. Mochizuki et al. [27, 28] reported that the footprint of the supraspinatus tendon on the greater tuberosity is much smaller than previously believed, and this area of the greater tuberosity is actually occupied by a substantial amount of the infraspinatus tendon. This may mean that during surgery, infraspinatus tendon repair rather than supraspinatus tendon repair may be closely associated not only with the footprint coverage at the greater tuberosity, but also with retear after surgery at the site.

The limitations of the present study were its retrospective cohort, short-term follow up, and small sample size, especially in group PS and APE in comparison with group AS. Further studies with longer follow-up and larger cohorts are needed to address these limitations. However, the strength of this study was that we clearly demonstrated that decreased active eternal range is a risk factor for large and massive tears, especially in PS and APE cuff tears.

## Conclusions

Although multivariate analysis failed to detect significant risk factor for retear in patients with anterosuperior large/massive cuff tears who undergo ARCR, it demonstrated that active external rotation less than 25° before surgery is a significant risk factor in those with posterosuperior large/massive tears. This study may help surgeons understand the results of arthroscopic surgery in patients with large/massive tears.

## Abbreviations

AERR: Active external rotation range
ARCR: Arthroscopic rotator cuff repair
AUC: Area under the curve
ERS: External rotation strength
FD: fatty degeneration
GFDI: The Global Fatty Degeneration Index
JOA: Japanese Orthopedic Association
MA: Muscle atrophy
MRI: Magnetic resonance imaging
RCTs: Rotator cuff tears
ROC: Receiver-operating characteristic
ROM: Range of motion
UCLA: The University of California, Los Angeles
